# Microstructure and Hardness of Hollow Tube Shells at Piercing in Two-High Screw Rolling Mill with Different Plugs

**DOI:** 10.3390/ma15062093

**Published:** 2022-03-11

**Authors:** Mikhail M. Skripalenko, Stanislav O. Rogachev, Boris A. Romantsev, Viacheslav E. Bazhenov, Mikhail N. Skripalenko, Andrei V. Danilin

**Affiliations:** 1 Department of Metal Forming, National University of Science and Technology MISiS, Leninski Prospect, 4, 119049 Moscow, Russia; boralr@yandex.ru (B.A.R.); tfsmn@yandex.ru (M.N.S.); danilinav@yandex.ru (A.V.D.); 2 Department of Physical Metallurgy and Physics of Strength, National University of Science and Technology MISiS, Leninski Prospect, 4, 119049 Moscow, Russia; csaap@mail.ru; 3 Casting Department, National University of Science and Technology MISiS, Leninski Prospect, 4, 119049 Moscow, Russia; v.e.bagenov@gmail.com

**Keywords:** screw piercing, two-high rolling mill, plug, hardness, grain size

## Abstract

AA6060 ingots were pierced in a two-high screw rolling mill (MISIS-130D) with guiding shoes (Mannesmann mill type). Three different plugs, i.e., a conventional entire plug, a plug with a cavity, and a hollow plug, were used for piercing. We established that the grain size decreases after piercing, by order of magnitude, compared to the initial non-pierced annealed bill, with a grain size of 100–400 μm, and the hollow shell grains are elongated along the piercing direction. The produced hollow shells had 30% higher hardness than the initial billet. The highest hardness values were obtained after piercing the conventional entire plug. The most uniform hardness distribution through the hollow shell’s volume was obtained after piercing the hollow plug. The cross and longitudinal section hardness measurements demonstrate that the hardness decreases from the outer surface to the inner surface of the hollow shells.

## 1. Introduction

One of the ways of producing hollow shells is piercing in a two-high screw rolling mill. This technique has a range of advantages. For instance, screw piercing has higher productivity than press piercing [1]. Different guiding tools are applied in two-high screw rolling mills to keep the billet in the deformation zone. The guiding tools could be guiding shoes, and such mills are called Mannesmann mills [2,3,4,5,6]. Guiding disks are applied in two-high mills of the Diescher type [7,8,9,10,11]. Several categories of investigation, depending on the research direction and estimated parameters, can be detected among publications concerning piercing in two-high screw rolling mills. One such category is the estimation of the loads and energy parameters of the screw piercing process [9,12,13,14], including the loads on the plug and rolls. One more trend is research on axial fracture, the so-called Mannesmann effect [15,16]. Quite a few publications, including [17,18], relate to computer simulations of piercing in two-high screw rolling mills, with the purpose of estimating a hollow shell’s stress–strain state. Some research is concerned with increasing the wear resistance of rolls and plugs, and choosing a material for forming tools [19].

Regarding the review of two-high screw rolling research publications, it is worth noting articles connected with hollow shell microstructure formation under different deformation regimes [20,21,22,23,24]. Publications [25,26] are about the influence of the shape and dimensions of the plug on the accuracy of the hollow shell dimensions. As a result of a review of the publications, no articles concerning the impact of the shape and dimensions of the plug on the hollow shells’ properties were found. The estimation of the specified impact is relevant because it allows recommendations on choosing a plug for piercing in two-high screw rolling mills to be designed, to obtain the most uniform hollow shell property distribution.

The research objective was to investigate the hardness and microstructure of aluminum alloy hollow shells after two-high screw rolling in a mill with guiding shoes, while using an entire plug, a hollow plug, and a plug with a cavity. The idea of using such plugs was to estimate how a difference in metal flow character influences microstructure and hardness. In the case of the traditional entire plug, metal only flows along the surface of the plug. In the case of the hollow plug, metal not only flows along the surface of the plug, but also in it. In the case of the plug with a cavity, there is metal flow along the surface of the plug and limited flow inside the plug. Regarding the flow inside the plug, it was found to be limited by the depth of the cavity.

## 2. Materials and Methods

### 2.1. Casting of Ingots

AA6060 aluminum alloy scrap was used as a raw material. Melting was realized using a clay graphite crucible in a resistance furnace. The alloy was treated with the ARSAL 2125 refining flux at 760 °C, and degassing was performed using hexachloroethane (C_2_Cl_6_) at the same temperature. The refined melt was kept for 15 min and poured into a mold at 740 °C. The mold was preheated up to 150 °C. The mold’s material was an A356 aluminum alloy. Ingots of 60 mm diameter and 210 mm height were cast. Before pouring, the mold was covered using Cillolin Al285 coating [25,26]. The AA6060 alloy was used for experiments because it has good technological plasticity. This alloy is a perspective material for manufacturing long length seamless tubes using tube rolling machines.

### 2.2. Two-High Screw Rolling

**Figure 1:**
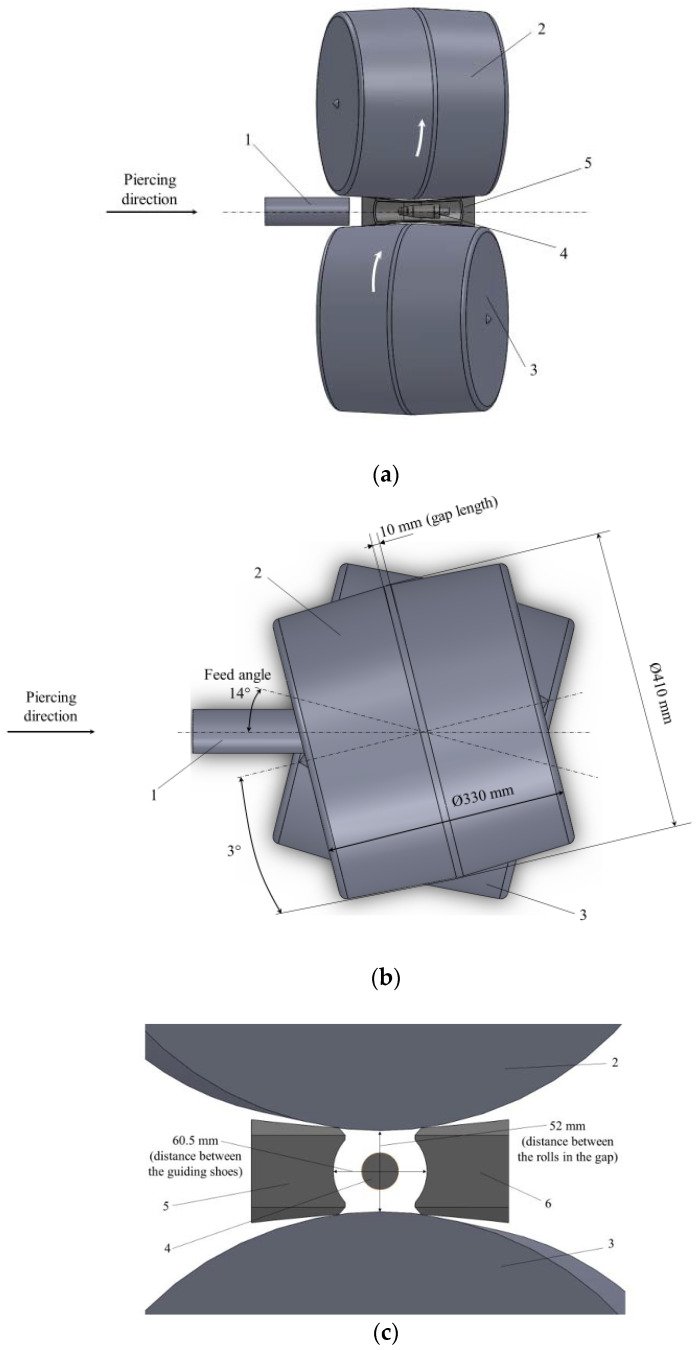
Screw piercing scheme in the MISIS-130D two-high mill: (**a**) side view, (**b**) top view, (**c**) gap cross-section; 1—billet, 2, 3—rolls, 4—plug, 5, 6—guiding shoes.

The ingots were annealed at 400 °C for 2 h. Billets were then subjected to hot plastic deformation piercing in a two-high screw rolling mill. Screw piercing of the annealed ingots was conducted using a MISIS-130D mill (The Electrostal Heavy EngineeringWorks JSC (“EZTM” JSC), Electrostal city, Moscow Region, Russian Federation) [25,26]. The pierced billet was extracted by two barrel-type rolls, and turned at 14 degrees of the feed angle. Modern two-high screw rolling mills, which are used for tube piercing, operate at 12–14 degree feed angles. That is why a 14 degree feed angle was set during experiments. The inclination angle of rolls was 0 degrees. The shoes were a guiding tool in the mill. The distance between the rolls in the gap (or draft) was 52 mm. The distance between the guiding shoes was 60.5 mm. Diameter reduction between the rolls in the gap, from 60 mm to 52 mm, was 13.3%, which is the diameter reduction commonly used for hot screw piercing of metal hollow shells. The gap section of the rolls was 410 mm, and the gap length on the rolls was 10 mm. The entry and exit tapers for the rolls were equal in length, and were 3°. The rolls’ rotational speed was 5.23 radians per second. The piercing scheme is presented in Figure 1. One of the guiding shoes is not shown in Figure 1a for convenience.

**Figure 2:**
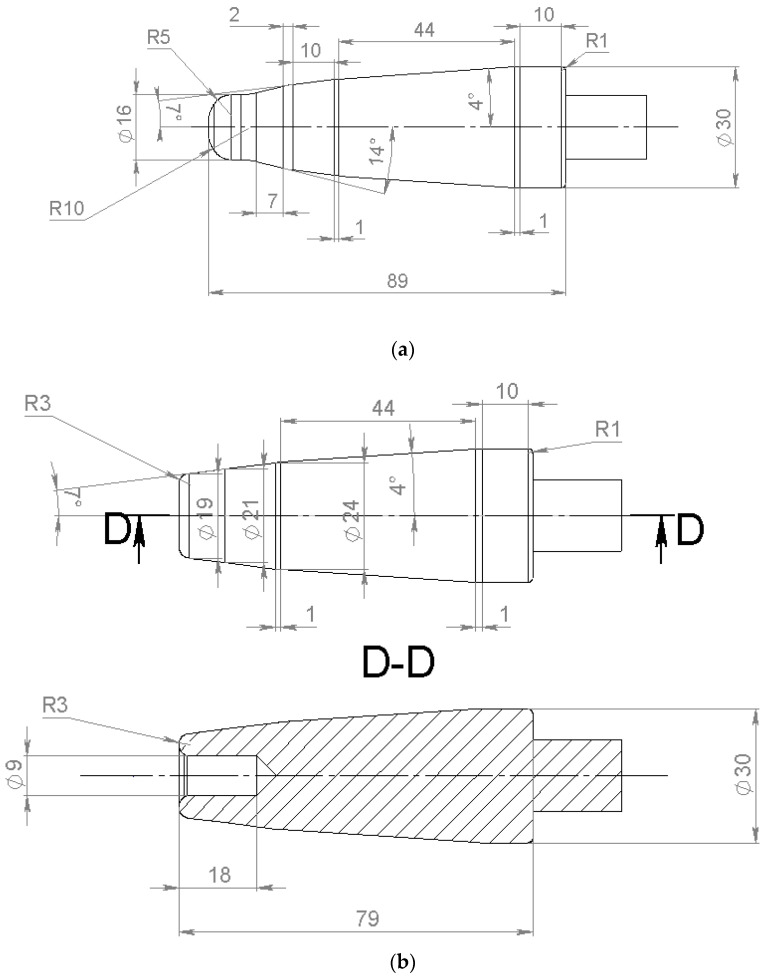

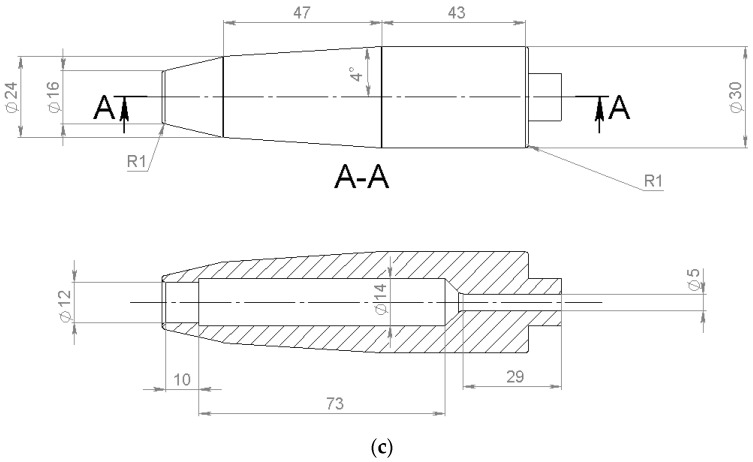
Plugs for piercing the ingots: the entire plug (**a**), the plug with a cavity (**b**), and the hollow plug (**c**).

Three plugs were used for piercing, namely, an entire plug, a plug with a cavity, and a hollow plug (Figure 2, all the units are “mm”). These plugs were developed in our previous research [26]. The calibrating section diameter of each plug was 30 mm. The hollow plug and the plug with a cavity were manufactured in concordance with the entire plug construction, maintaining length and obliquity at each section of the plugs. The entire plug’s nose was 30 mm in front of the draft, the edge of the plug with a cavity was 20.6 mm in front of the draft, and the edge of the hollow plug was 15.5 mm in front of the draft.

Sections of length 30 mm were cut from the hot top of the ingots before piercing. The remaining part was used to center the plugs while piercing, as was conducted in our previous experimental work [25,26].

### 2.3. Microstructure Investigation

**Figure 3:**
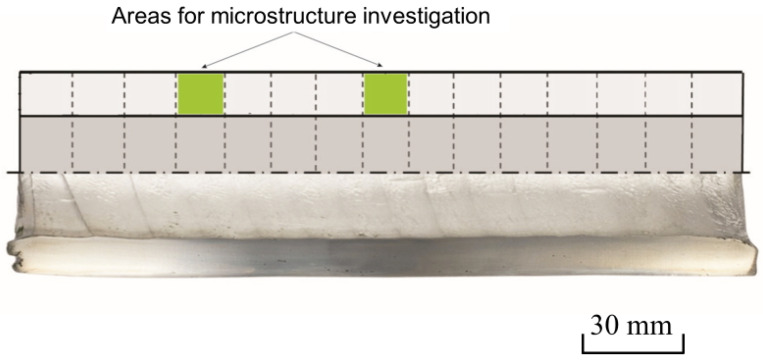
Location scheme of the samples used for the microstructure investigation in the longitudinal section of the hollow shell.

The investigation of the hollow shells’ microstructure was performed using an Axio Scope A1 optical microscope (Carl Zeiss Microscopy GmbH, Jena, Germany), with Tixomet software. Magnification was ×50–×1000; etching was performed using HF:H_2_O = 1:20. Each hollow shell was cut into 15 equal pieces before the microstructure investigation. The microstructure of the hollow shells’ cross-sections was studied at ½ and ¼ hollow shell length at a distance from the back edge of the hollow shell (Figure 3). The linear intercept method was used for the grain size estimation.

### 2.4. Hardness Measurement

**Figure 4:**
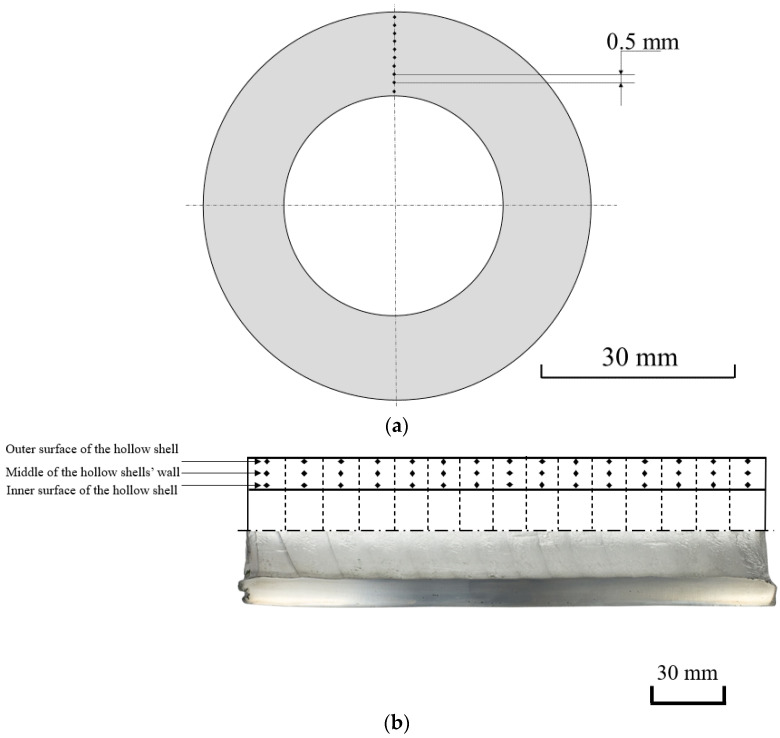
Microhardness measurement scheme in the cross-section at the stationary stage of piercing (**a**) and the longitudinal section (**b**) of the hollow shell.

Vickers microhardness was measured using a MicroMet 5101 (Buehler, Leinfelden-Echterdingen, Germany) (3 N load, 10 s holding). The microhardness measurements were obtained in the hollow shell cross-section at the stationary piercing stage (Figure 4a). The areas for the measurements were located 0.5 mm apart from each other. For each area, three measurements were performed, and the mean values were calculated. One specimen was studied for each of the piercing cases to estimate hardness in the cross-section of the hollow plugs at the stationary stage. The microhardness was also measured in the longitudinal section. The procedure was repeated for each of the 15 pieces near the outer and inner surfaces of the hollow shells, and in the middle of the hollow shell wall. Three measurements were performed for each area (points in Figure 4b), and the mean values were calculated. The microhardness measurement of the non-pierced billet (in annealed condition) was conducted along the radius in 5 points with a 7.5 mm step.

## 3. Results and Discussion

**Figure 5:**
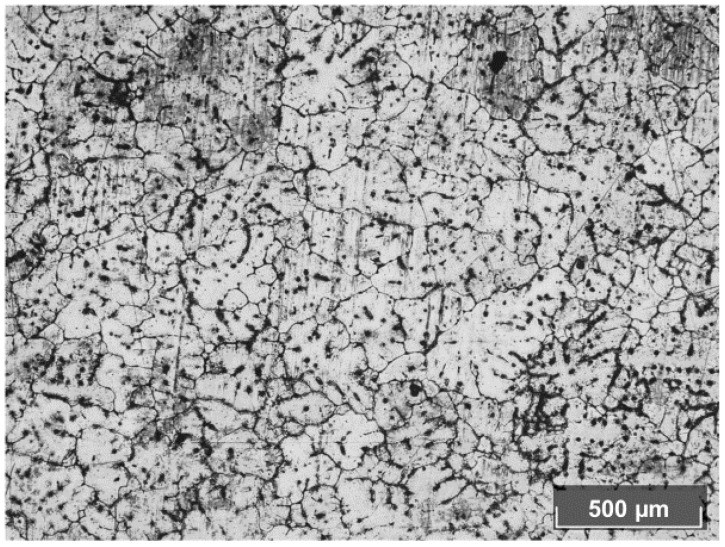
Annealed aluminum alloy billet structure (before piercing).

The initial non-pierced billet (2 h annealing at 400 °C) has a coarse-grained structure, with a grain size of 100–400 μm (Figure 5). The initial non-pierced billet’s hardness values are within the 30–36 HV limits, with the mean value being 33 HV.

**Figure 6:**
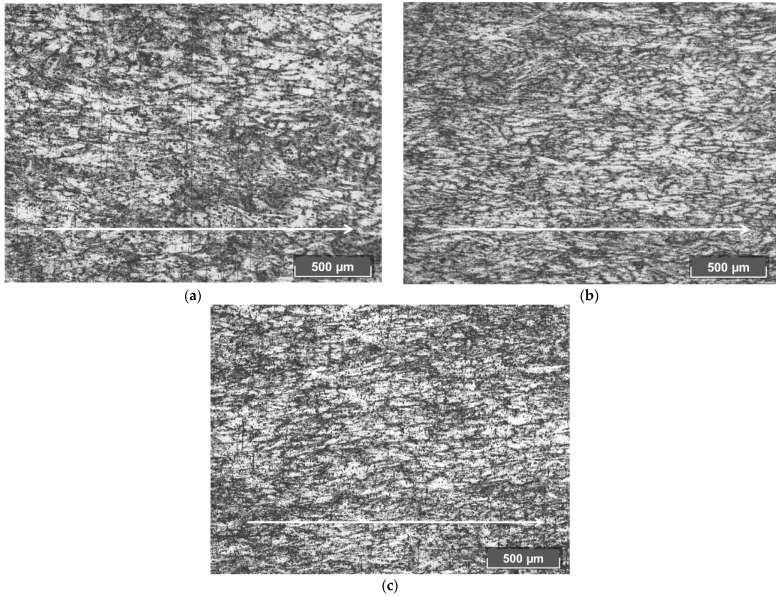
Hollow shell’s longitudinal section microstructure from the middle of the hollow shell’s wall after piercing by the entire plug (**a**), the plug with a cavity (**b**), and the hollow plug (**c**) (the arrows show the piercing direction).

The grain size in the hollow shells’ structure decreases by order of magnitude com-pared to the non-pierced billet. In the longitudinal section, grains elongated along the piercing direction are observed (Figure 6). Such elongated grains (subgrains) form a fibrous structure, typical of the formed metal. The structure near the hollow shells’ surface is more inhomogeneous than in the middle of the wall. Near the surface, the boundaries of the structural elements are strongly lacerated and very poorly distinguishable, which is apparently associated with a high density of crystal defects. In the middle of the hollow shells’ wall, the subgrain boundaries are better distinguished. The elongated subgrains are mostly 20–55 μm in size. The microstructure of the hollow shells obtained by entire plug piercing and hollow plug piercing is similar, and it differs from the hollow shell microstructure produced by the plug with cavity piercing. In the latter case, subgrains have more regular boundaries that are easier to distinguish.

**Figure 7:**
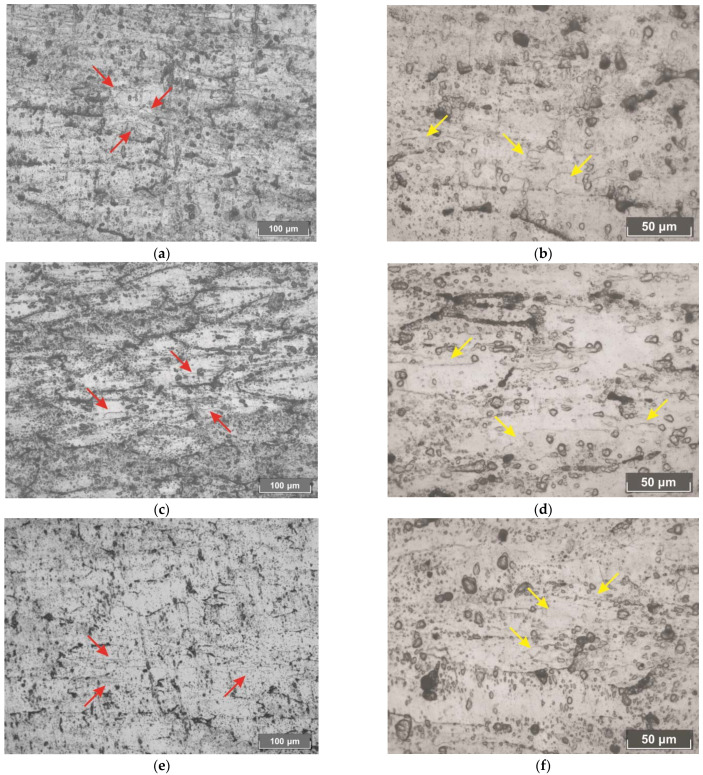
Dynamically recrystallized grains in the longitudinal section of the hollow shells after piercing by the entire plug (**a**,**b**), the plug with a cavity (**c**,**d**), and the hollow plug (**e**,**f**).

The elongated chain of dynamically recrystallized small grains, 5–10 μm in size with rough edges, is observed near the subgrains’ boundaries (Figure 7). These chains are identified with arrows in Figure 7.

**Figure 8:**
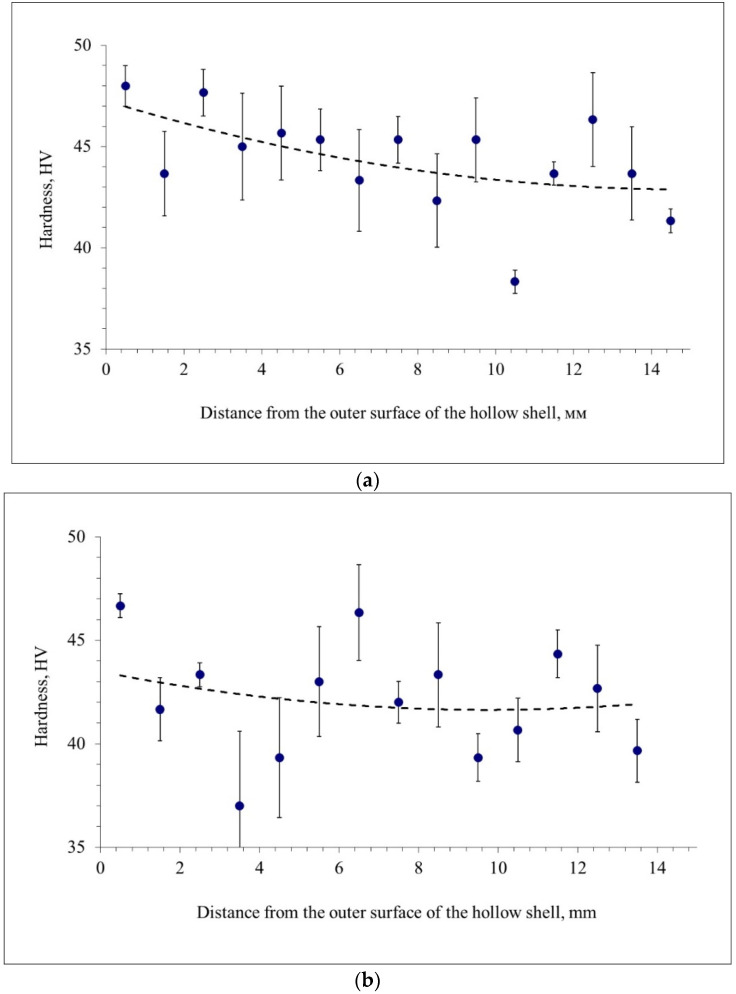

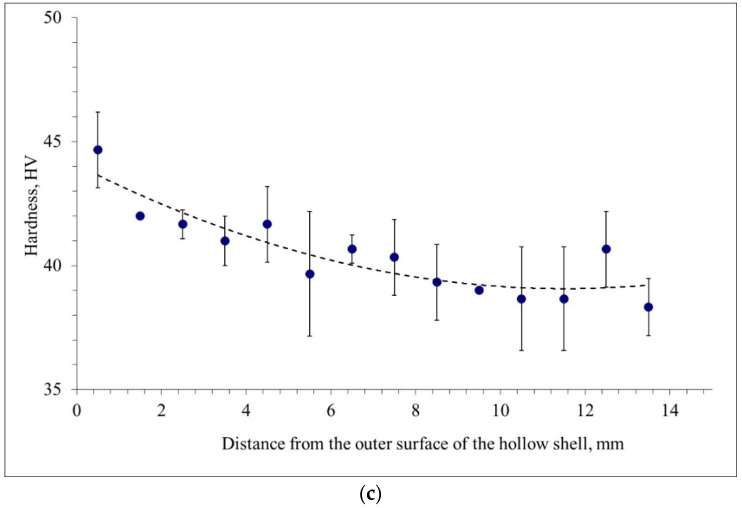
Hardness variation along the wall of the hollow shells pierced by the entire plug (**a**), the plug with a cavity (**b**), and the hollow plug (**c**).

The hardness variation along the walls of the hollow shells pierced by the different plugs is given in Figure 8. In other words, Figure 8 illustrates how hardness changes in the cross-section of the hollow shells at the stationary stage, according to the measuring scheme in Figure 4a. The markers in Figure 8 indicate the average values, and error bars are given next to them. It is worth noting that the average value of hollow shell diameter was 60.6 mm after entire plug piercing, 61 mm after plug with a cavity piercing, and 60.1 mm after hollow plug piercing. The average value of wall thickness was 14.7 mm after entire plug piercing, 15.1 mm after plug with a cavity piercing, and 13.7 mm after hollow plug piercing.

According to Figure 8, the hardness for all hollow shells tends to decrease from the outer surface to the inner surface of the hollow shell. This decreasing tendency is also shown by the trend lines (governed by 2nd power polynomials). The mean values, the range, and the variance of hardness were calculated for quantitative estimation (Table 1). According to Table 1, the hardness is most uniformly distributed in the cross-section of the hollow shell produced by hollow plug piercing. In this case, the variance is half as much as that discovered when using the entire plug or the plug with a cavity. The highest hardness values in the cross-section of the hollow shell are those for the entire plug piercing.

The hardness fluctuations may be due to the presence of deformed areas with a high concentration of crystal defects that have higher density. They could also be due to dynamically recrystallized areas that have low hardness values. At the same time, the structure type after piercing has little effect on the alloy’s microhardness. One of the possible reasons for this is that the subgrain structure is formed because of the piercing. It is known that the subgrain size affects the alloy’s strength less than the grain size [27].

**Figure 9:**
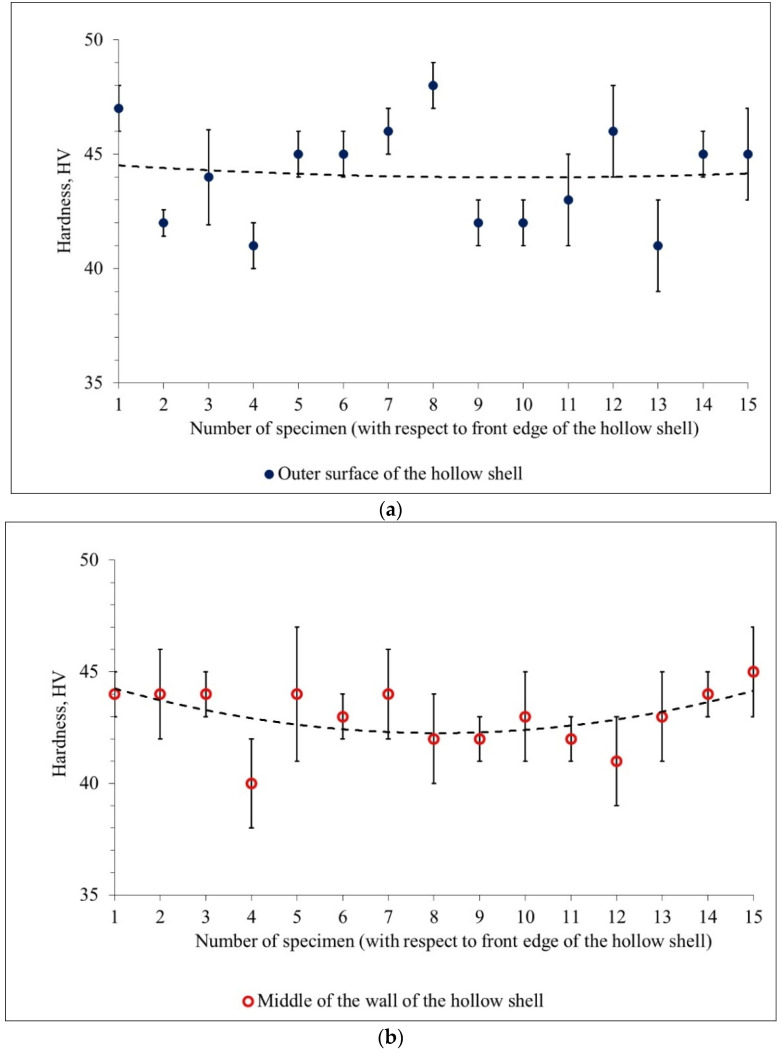

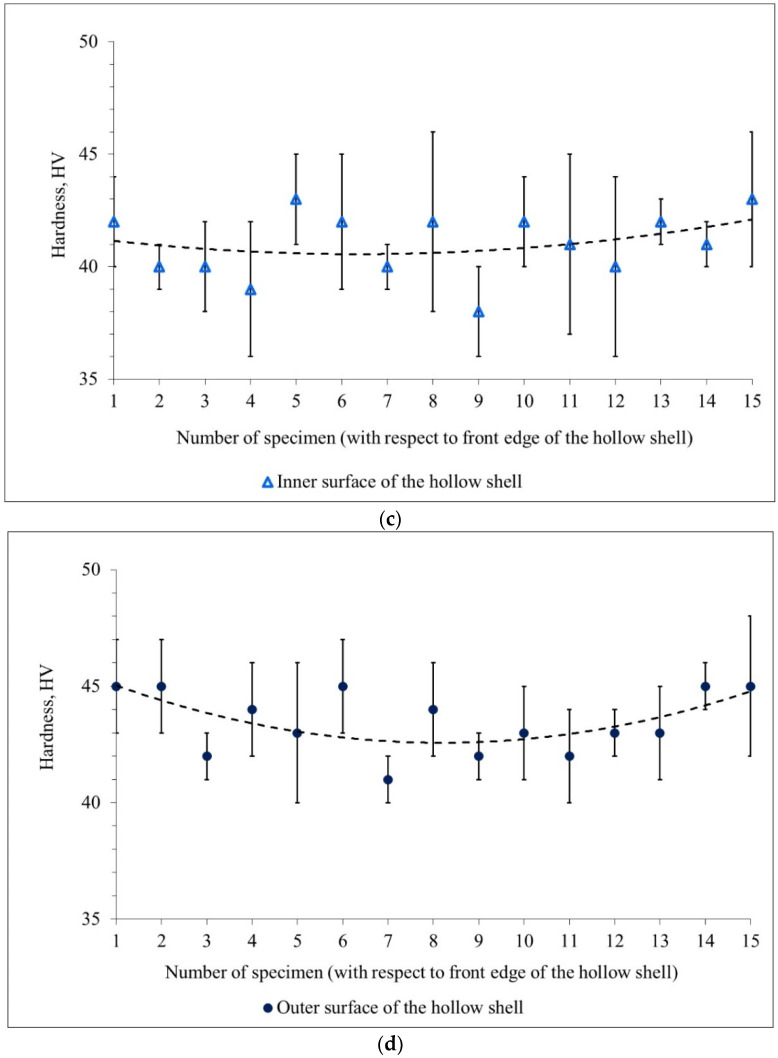

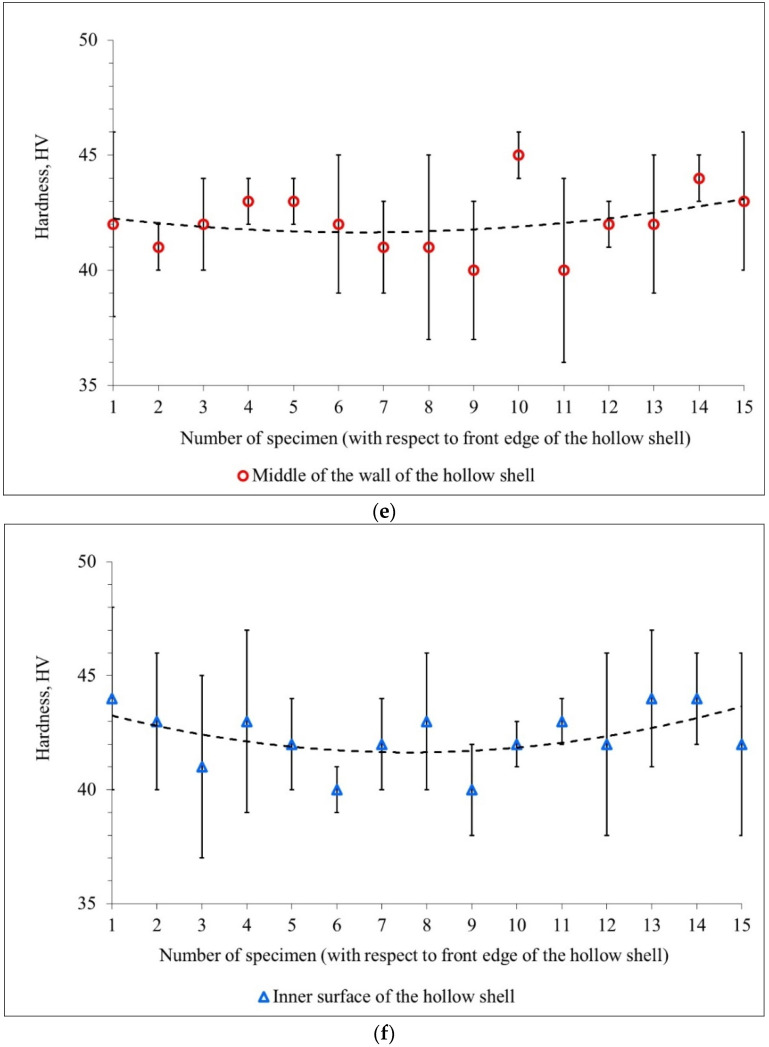

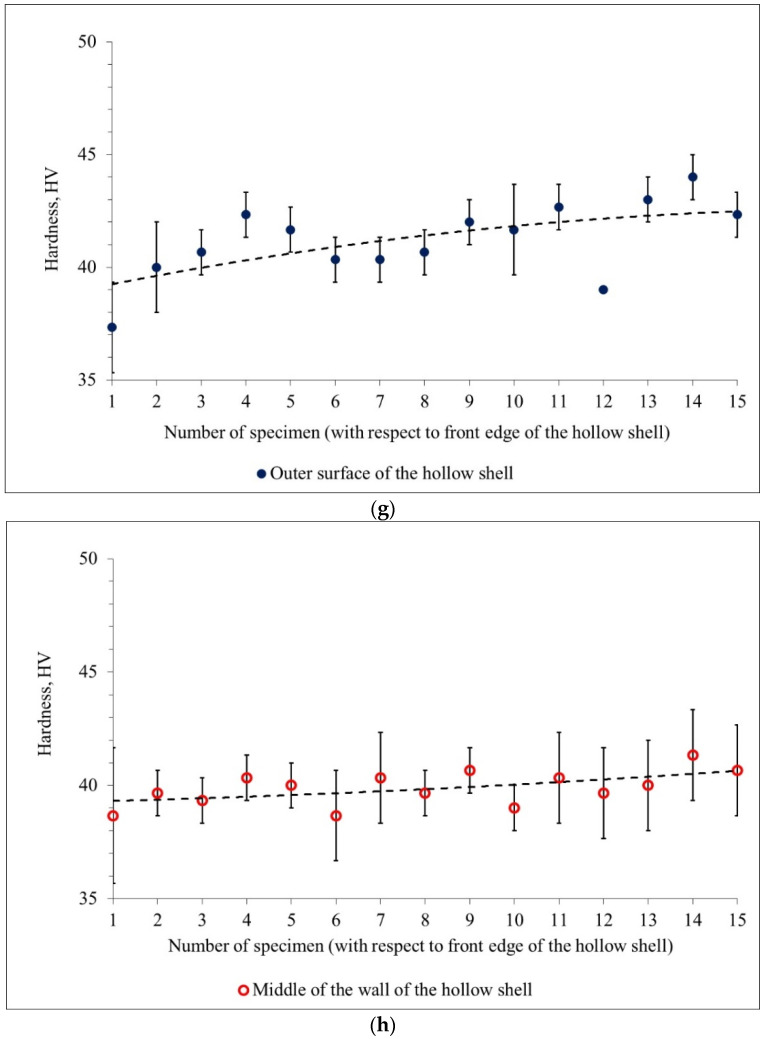

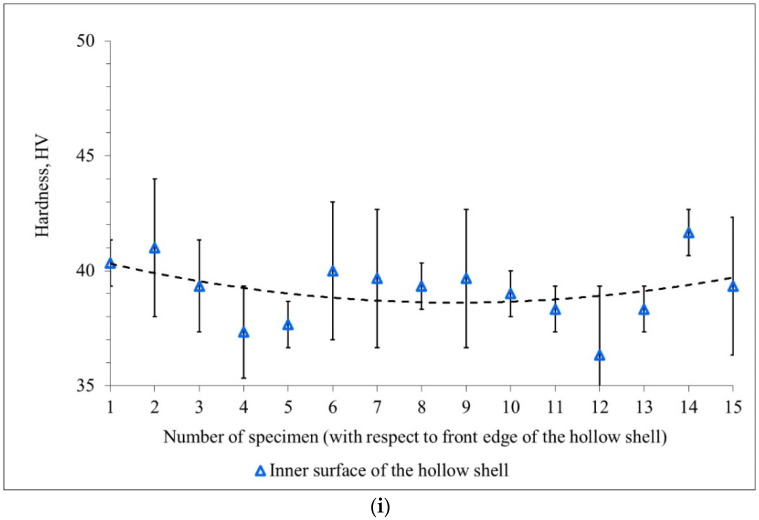
Hardness changing in the longitudinal section of the hollow shells pierced by the entire plug (**a**–**c**), the plug with a cavity (**d**–**f**), and the hollow plug (**g**–**i**).

The hardness measurements in the longitudinal section are presented in Figure 9. These measurements were performed according to the scheme in Figure 4b. The markers in Figure 9 represent the average values, and error bars are given next to them. For the majority of the investigated specimens, the outer surface hardness points lie above the middle of the wall hardness points, which, in turn, lie above the inner surface hardness points. The data in Figure 9 confirm the trend revealed by Figure 8 for the cross-section; namely, the hardness decreases from the outer surface to the inner surface of the hollow shells. The trend lines in Figure 9b–f reveal the tendency for the hardness to be higher at the edges of the hollow shells (non-stationary stage) than in the middle of the hollow shells (stationary stage). However, visually, the edge hardness values are the maximum at the front edge in Figure 9a (entire plug piercing), at both edges in Figure 9b (entire plug piercing), at the front edge in Figure 9d (plug with a cavity piercing), and at the front edge in Figure 9f (hollow plug piercing).

The quantitative estimation of the data shown in Figure 9 was carried out to estimate the uniformity of hardness distribution in the longitudinal section (Table 2). The hardness variance values for the hollow plug piercing (2.4 HV^2^) and for the plug with a cavity piercing (2.1 HV^2^) are 1.8 and 2.1 times less than the hardness variance value for the entire plug piercing (4.5 HV^2^), respectively.

The mean values of hardness in the longitudinal section of the hollow shells (Table 2) do not differ from those for the cross-section of the hollow shells; namely, the highest hardness values are for the hollow shells produced by the entire plug, with slightly lower values for the hollow shells produced by the plug with a cavity, and the lowest values for those produced by the hollow plug piercing. Notably, the mean values of hardness for all the hollow shells do not differ by more than 4 HV.

It is worth noting that the mean value of hardness increases compared to the mean value of the non-pierced billet. This increase is 33% for the entire plug piercing, 28% for the plug with a cavity piercing, and 21% for the hollow plug piercing.

## 4. Conclusions

The piercing of AA6060 aluminum alloy ingots was conducted in a two-high screw rolling mill MISIS-130D with guiding shoes. A traditional entire plug, a plug with a cavity, and a hollow plug were used for piercing. The study made it possible to establish the following:

1. The average grain size of the hollow shells decreases by order of magnitude (up to 20 times) compared to the non-pierced billet. Notably, the hollow shells’ grains (subgrains) are elongated in the piercing direction. The elongated chains of dynamically recrystallized grains, 5–10 μm size, with rough edges, are visible near the subgrain boundaries.
2. The hardness for all hollow shells decreases from the outer surface to the inner surface. The mean value of hardness is the highest after the entire plug piercing (44 HV), followed by the plug with a cavity piercing (42–43 HV), then the hollow plug piercing (40–41 HV), and the mean value is the lowest for the non-pierced billet (33 HV).
3. The hardness measurements for the cross and longitudinal sections of the hollow shells reveal a common trend; namely, the hardness decreases from the outer surface to the inner surface of the hollow shell.
4. The experiments demonstrated that the traditional entire plug allows the achievement of the highest values of hardness and, hence, the highest material strength of all three investigated piercing cases. The hollow plug provides the most uniform hardness distribution through the hollow shells’ volume. Hence, it is possible to deduce that when metal only flows between the rolls and plug (as in the entire plug case), one can gain higher hardness and, hence, strength, but with a decrease in hardness distribution uniformity. Whereas, when metal not only flows between the rolls and plug, but also inside the plug, the uniformity of hardness distribution is higher, but the hardness values are less compared to those achieved with the traditional entire plug. For the plug with a cavity, the outcome is somewhere between those for the entire plug and the hollow plug. The estimated effect of plug construction on the hollow shell’s properties is important. Even though a hollow shell is a semi-finished product, it sufficiently defines the properties of the final seamless tube, which is produced from the hollow shell by an elongation process. Hence, plug construction is sufficient, in terms of defining the quality of the final seamless tubes.

## Figures and Tables

**Table 1:** The hardness distribution indexes in the cross-section of the hollow shells at the stationary stage of piercing.

	Range, HV	Mean Value, HV	Variance, HV^2^
Entire plug	11	44.3	8.1
Plug with a cavity	12	42.1	9.8
Hollow plug	9	40.5	4.3

**Table 2:** The hardness distribution indexes in the longitudinal section of the hollow shells.

	Range, HV	Mean Value, HV	Variance, HV^2^
Entire plug	10	42.7	4.5
Plug with a cavity	5	42.6	2.1
Hollow plug	8	40.0	2.4

## Data Availability

Not applicable.
